# Retrospective review of immobilization vs. immediate resumption of activity in patients with Oligoarticular juvenile idiopathic arthritis following knee injections

**DOI:** 10.1186/s12969-019-0339-0

**Published:** 2019-07-12

**Authors:** Elaine R. Flanagan, Heather Benham, Janet Figueroa, Janille Diaz, Jenna Tress, David D. Sherry

**Affiliations:** 1 Children’s Healthcare of Atlanta/Emory University, Atlanta, GA USA; 2 Texas Scottish Rite Hospital, Dallas, TX USA; 3 Children’s Hospital of Philadelphia, Philadelphia, PA USA

**Keywords:** Oligoarticular juvenile idiopathic arthritis, Knee injection, Joint injection, JIA splint

## Abstract

**Background:**

Intraarticular corticosteroid injection (IACI) is one of the most common treatments in oligoarticular Juvenile Idiopathic Arthritis (JIA). Activity recommendations following injection vary, as there are no published studies on splinting JIA patients post-IACI (splinting is a form of rest). Texas Scottish Rite Hospital for Children (TSRH) splints patients post-IACI for 24 h while The Children’s Hospital of Philadelphia (CHOP) does not. The aim of this study was to compare the number of cases of recurrent arthritis following IACI between these two post-injection practices.

**Methods:**

Data were retrospectively collected at CHOP and TSRH. Patients diagnosed with oligoarticular JIA according to International League of Associations for Rheumatology (ILAR) criteria (2nd revision, 2001) between 2008 and 2010 were included. Bivariate analysis (Wilcoxon rank-sum tests, chi-squared tests) was run to assess differences in outcomes by site. Inverse probability of treatment weighted Cox regression was employed to adjust for site differences.

**Results:**

The population at TSRH was younger than at CHOP (*p* < 0.05) and had more whites (*p* = 0.03). Disease duration was significantly longer at TSRH than at CHOP (0.40 vs. 0.74 years, *p* = 0.014). More children were on biologics at the time of injection at CHOP (p < 0.05). The baseline physician global (*p* < 0.001) was higher at CHOP, as was the joint disease severity (p < 0.001). CHOP had fewer reoccurrences of knee arthritis compared to TSRH: 26% vs 38% (*p* = 0.14).

**Conclusions:**

The baseline populations were different in that the TSRH group had more whites and Hispanics, were younger and, perhaps, had less severe disease than CHOP. Patients treated with post-injection splinting had a trend toward more arthritis reoccurrence (38% vs. 26%, p = 0.14). Splinting is not clearly beneficial post-injection.

**Trial registration:**

This is an observational study, so it is not applicable.

**Electronic supplementary material:**

The online version of this article (10.1186/s12969-019-0339-0) contains supplementary material, which is available to authorized users.

## Background

Oligoarticular Juvenile Idiopathic Arthritis (JIA) is defined as arthritis affecting four or less joints, lasting six weeks or more, and onset prior to the age of 16 years [1]. It affects 36 per 100,000 children and accounts for 50 to 80% of all JIA cases [2]. In addition to the use of nonsteroidal anti-inflammatory drugs, intra-articular corticosteroid injection (IACI) is one of the most common treatment modalities in oligoarticular JIA [3–5]. Introduction of steroid directly into an affected joint provides reduction of inflammation [4], relief of pain, increased mobility [6], and can help prevent leg length discrepancy when a single knee is involved [7]. Many studies have examined the efficacy of IACI in children with arthritis. Padeh et al. found that approximately 82% of joints injected in oligoarticular JIA patients resulted in remission [6]. Papadopoulu looked at all subtypes of JIA patients receiving IACI and 34% went into remission for 0.9 years [8]. 67 to 82% of injected joints showed resolution of arthritis findings six months post-procedure in additional studies [9–11].

Despite the widespread use of local steroid injections in the treatment of arthritis, the recommendations following the procedure vary greatly [12]. Post-injection protocols range from complete bedrest to immediate resumption of normal activity. A more intermediate modality may be the use of a splinting device for a 24–48 h period. There have been no studies in the pediatric population, more specifically in those with juvenile idiopathic arthritis, examining post-joint injection regimens. Most joint injection studies in this population, which primarily examine efficacy, make the recommendation of rest or decreased activity for the 24-h period afterwards as part of their procedure protocol [1, 8, 10, 11, 13–16]. Breit et al. mention that the 24-h rest period could involve restriction to bed, wheelchair, or splint but they do not mention any specifics [17]. Several studies mention a rest period of 72 h [18, 19] while others do not outline any specific post-joint injection procedure [9, 20].

The issue of splinting or rest following IACI has been examined to a limited extent in the adult arthritis population. McCarty et al. state the rationale for resting a joint after injection is threefold: 1) to minimize leakage of steroid crystals back through the needle track, 2) to provide time for repair of inflammatory tissue damage and 3) to minimize loss of soluble steroid into the systemic circulation [21]. Winfield at al. showed that both bed rest and splinting reduced the uptake of yttrium-90 from the knee following intra-articular injection suggesting that these types of restrictions post-IACI may similarly reduce systemic uptake of the corticosteroid [22]. Neustadt adds that post-injection rest can increase the duration of clinic benefit and diminish potential cartilage damage [23–25].

Whether this practice is beneficial seems to depend, to some extent, on which joint is injected. Chakravarty et al. found that adults treated with IACI for knee synovitis who were assigned to bed rest for 24 h had greater improvement in pain and stiffness, 50 ft walking test, knee circumference, and CRP for 6 months [26]. In addition, Weitoft et al. found greater reduction in serum cartilage matrix protein (a marker of cartilage turnover) in RA patients after 24 h of bedrest post-IACI [27]. The two studies examining this issue in upper extremity joint (wrist, elbow) IACI found increased relapse rates in the immobilization groups (did not reach statistical significance); although, there were not differences in multiple secondary outcome measures [28, 29]. It is suggested from this limited research in adults that some clearly defined post-IACI procedure, such as a defined rest period, may improve outcomes, specifically for the knee.

There are no published studies comparing specific post-IACI procedures, such as splinting or bedrest, in oligoarticular JIA patients. JIA patients treated at Texas Scottish Rite Hospital for Children (TSRH) with IACI for knee synovitis are immobilized for 24 h post-procedure, to encourage rest, while those at The Children’s Hospital of Philadelphia (CHOP) are instructed to avoid sports the day of injection. The aim of this retrospective study was to compare the number of cases of recurrent arthritis and time to recurrent arthritis among oligoarticular JIA patients treated with IACI of the knee between institutions.

## Methods

**Subjects.** The source population for this study was children and adolescents with a diagnosis of oligoarticular JIA with a knee injection occurring between 2008 and 2010. Patients met ILAR criteria (2nd revision, 2001) at CHOP and TSRH rheumatology clinics. Each subject was followed for 24 months from the date of injection or December 31, 2012, whichever was sooner. The following were the inclusion criteria: subjects aged 0 to 18 years, diagnosis of oligoarticular JIA, knee IACI performed between 2008 and 2010, and follow up for 2 years after joint injection. The exclusion criteria were as follows: concurrent disease that would influence the outcome of the IACI (i.e. inflammatory bowel disease, pigmented villonodular synovitis, osteochondritis dessicans, trauma, infection, neoplasm, sarcoidosis, systemic lupus erythematosus, or periodic fever syndromes), lack of resolution of arthritis post-injection, or.missing data. 12 patients had arthritis which did not resolve, so we excluded them. 1 patient had missing data about whether arthritis recurred and the number of months to recurrence.

**Programs.** The standard practice at CHOP for knee injections is to instruct patients to refrain from sport activities the day of IACI. At TSRH, patients who have a knee injected are placed in a splint for 24 h post-IACI and advised to rest.

### Measures of interest

#### Clinical characteristics

The following clinical characteristics were recorded from the time of IACI: sex, age, race, ethnicity, age of onset of JIA, age of knee symptom onset, presence of iritis, ESR, ANA, RF, HLA-B27, Childhood Health Assessment Questionnaire (CHAQ), Parent/Patient global for pain, Physician global for disease activity and total number of active joints. Disease duration was calculated as follows: age at study - (age at diagnosis or knee symptom start, whichever was earlier).

#### Joint injection dose

Triamcinolone hexacetonide (Aristospan®) was the primary corticosteroid used for injections. When not available, Triamcinolone acetonide (Kenalog®) was used as an alternative. The treating clinician determined the injection dose. Dose per knee was recorded and mg/kg dose was calculated for each injection.

#### Joint exam

All patients had a complete joint exam conducted prior to the injection, as well as at clinic follow up. This exam included assessment of joint swelling, joint tenderness, and joint range of motion (ROM). Each joint component was graded on a scale from zero (none) to four (severe). The determination of active arthritis was based on clinical exam: swelling (not due to inactive synovitis or bony enlargement) or if no swelling, limited ROM + heat, pain, or tenderness [30].

#### Medications

Prescriptions for intraocular steroids, corticosteroids, disease-modifying antirheumatic drugs (DMARDs), and biologics were recorded at the time of injection, as well as post-injection. Non-steroidal anti-inflammatory drugs (NSAIDs) were recorded at any time.

### Analysis

All statistical analysis was conducted using SAS 9.4 (Cary, NC) and R version 3.4.1. Demographic information and baseline characteristics for each center were summarized by frequencies and percentages for categorical variables (e.g. sex, race, ethnicity, ANA, RF, HLA-B27 status) and by median and interquartile range (IQR) for continuous variables. Wilcoxon Rank-Sum tests and Chi-Square/Fisher’s Exact Tests were conducted to determine differences by hospital center. Rank-based standardized mean differences (SMD) were calculated to determine the effect sizes and to assess which variables were imbalanced between the two sites using a 0.25 cut-off [31]. Inverse probability of treatment weighting (IPTW) using the average treatment effect (ATE) weight will be employed to address these imbalances based on observed measured differences (SMD > 0.25) [32]. Correlations between variables that were imbalanced were checked (*p* < 0.05 for Pearson correlations) and any collinear variables (diagnosis age correlated with height, weight, and age of disease onset; disease duration correlated with height, weight, and age of knee symptom start and age at study, total joints injected correlated with total joints active) were excluded from the propensity score analysis. ATE weights were estimated using the *twang* package in R. Weights were subsequently stabilized and trimmed at the 90th percentile (untrimmed weights were also assessed), then used in an adjusted Cox model [33]. Balance between the two sites was assessed using a number of diagnostic criteria including pre-post weighted SMD comparisons (Additional file 1: Figure S1) [34]. Any covariate that failed to achieve balance after propensity weighting (SMD > 0.25) was additionally adjusted for in the Cox model. Statistical significance was determined at the 0.05 level.

## Results

Demographic information is summarized in Table 1. No significant differences between centers were observed for sex or ethnicity. The population at TSRH was significantly younger at disease onset and at the time of knee injection (median of 5.6 years vs. 7.5 years, *p* = 0.0416, SMD = 0.32) and was more likely to be white (85% vs. 94%, *p* = 0.0327, SMD = 0.33). Disease duration was significantly longer at TSRH than at CHOP (0.40 vs. 0.74 years, *p* = 0.0141, SMD = 0.44).

**Table 1:** Population Characteristics, *N* = 167 (single injection)

N(%) or Median (25th–75th)					
	All (N = 167)	CHOP (*N* = 72)	TSRH (*N* = 95)	*P*-value	SMD
Sex - Female	136 (81%)	58 (81%)	78 (82%)	0.80	0.04
Age, years	6.7 (3.8–10.5)	7.5 (4.8–11.3)	5.6 (3.5–9.8)	**0.0416**	0.32
Race				0.06	0.36
Black/AA	5 (3%)	2 (3%)	3 (3%)		
White	149 (90%)	60 (85%)	89 (94%)		
Other	12 (7%)	9 (13%)	3 (3%)		
White (vs. nonwhite)	149 (90%)	60 (85%)	89 (94%)	**0.0327**	0.33
Ethnicity – Hispanic (vs not)	25 (15%)	8 (11%)	17 (18%)	0.25	0.19
Symptom Age Start, years^a^	4.6 (2.3–8.7)	5.7 (3.8–9.8)	3.9 (2.1–8.2)	**0.04333**	0.35
Knee Symptom Age Start, years^b^	5.6 (3.2–9.7)	6.3 (3.5–10.1)	5.0 (2.9–9.2)	0.43	0.14
Diagnosis age	4.0 (2.5–6.8)	4.8 (2.5–7.5)	3.6 (2.5–6.0)	0.15	0.22
Disease duration, years^c^	0.58 (0.29–2.71)	0.40 (0.22–1.32)	0.74 (0.34–3.09)	**0.0141**	0.44
Systolic Blood Pressure	106.0 (101.0–115.5)	109.0 (102.0–118.5)	106.0 (100.0–114.0)	0.27	0.19
Diastolic Blood Pressure	63.0 (57.0–66.0)	63.0 (59.0–66.0)	62.0 (57.0–66.0)	0.38	0.15
Iritis – Yes^d^	4 (4%)	1 (2%)	3 (5%)	1.00	0.11

*Wilcoxon Rank-Sum tests or Chi-squared/Fisher’s Exact Tests between CHOP and TSRH sites, alpha = 0.05

Missing:

^a^Symptom age start, *n* = 30 missing

^b^Knee Symptom age start, *n* = 33 missing

^c^Disease duration, *n* = 38 missing

^d^Iritis, *n* = 59 missing (35%)

SMD: standardized mean difference. SMD > 0.25 (25%) indicates an imbalance between our groups, *P* < 0.05 is considered statistically significant (shown in bold)

Clinical information is presented in Table 2. Patients at CHOP were more likely to have two or more active joints at the time of knee injection (58% vs. 37%, *p* = 0.0058, SMD = 0.44). Significant differences were also observed in the initial joint exams of the involved knee (see Table 2). CHOP patients had more joint swelling, joint tenderness, and limitation of joint range of motion (*p* < 0.05, SMD ~ 0.35–0.94). TSRH patients reported more pain than at CHOP according to the pain assessment scale rating (*p* = 0.0081, SMD = 0.56). The physician global scores were higher at CHOP (median MD global score of 2 vs. 1, *p* < 0.0001, SMD = 1.80).

**Table 2:** Clinical Characteristics, N = 167 (single injection)

N(%) or Median (25th–75th)					
	All (N = 167)	CHOP (N = 72)	TSRH (N = 95)	P-value*	SMD
Joint swelling					
> 1 (vs 0)	161 (96%)	69 (96%)	92 (97%)	1.00	0.05
Joint tenderness					
> 1 (vs 0)	30 (18%)	27 (38%)	3 (3%)	**< 0.0001**	**0.94**
Joint range of motion					
> 1 (vs 0)	95 (57%)	34 (47%)	61 (64%)	**0.0281**	**0.35**
Total joints active				**0.0046**	0.58
1	90 (54%)	30 (42%)	60 (63%)		
2	58 (35%)	28 (39%)	30 (32%)		
3	13 (8%)	8 (11%)	5 (5%)		
4	4 (2%)	4 (6%)	0		
5	0	0	0		
6	2 (1%)	2 (3%)	0		
2+ Joints Active (vs 1 only)	77 (46%)	42 (58%)	35 (37%)	**0.0058**	0.44
Pain Assessment (1–10)^a^	1 (0–4)	0 (0–4)	2 (0–4)	**0.0081**	0.56
CHAQ Score^b^	0.125 (0–0.50)	0.125 (0–0.125)	0.125 (0–0.50)	0.39	0.28
MD Global Score^c^	1(1–2)	2 (2–4)	1 (1–2)	**< 0.0001**	1.80
ANA status–Positive	108 (66%)	39 (55%)	69 (74%)	**0.0099**	0.41
RF status– Positive	4 (3%)	2 (3%)	2 (2%)	1.00	0.05
HLA-B27 status–Positive	14 (13%)	5 (11%)	9 (14%)	0.68	0.08
ESR^d^	14 (8–23)	12 (5–28)	14 (8–21.5)	0.48	0.14
On injection DMARDs	29 (17%)	12 (17%)	17 (18%)	0.84	0.03
On injection biologics	7 (4%)	6 (8%)	1 (1%)	**0.0432**	0.35
On NSAIDs	109 (65%)	49 (68%)	60 (63%)	0.51	0.10
Total joints injected				**0.0002**	0.60
2+	53 (32%)	34 (47%)	19 (20%)		
1	114 (68%)	38 (53%)	76 (80%)		
Steroid injection – Aristopan (vs. Kenalog)	156 (94%)	66 (92%)	90 (96%)	0.33	0.12
Knee injection dose (mg)	10 (2–20)	10 (2–20)	10 (2–20)	**0.0132**	0.40
Knee injection dose (mg/kg)	0.33 (0.10–0.78)	0.43 (0.13–0.90)	0.27 (0.10–0.72)	0.26	0.19

*Wilcoxon Rank-Sum tests or Chi-Squared/Fisher’s Exact Tests between CHOP and TSRH sites, alpha = 0.05

^a^Pain Assessment is missing for 42% (*n* = 70) of the data

^b^CHAQ score missing for 56% (*n* = 94) of the data

^c^MD Global score missing for 46% (*n* = 77) of the data

^d^ESR score missing for 31% (*n* = 52) of the data

SMD: standardized mean difference. SMD > 0.25 (25%) indicates an imbalance between groups, *P* < 0.05 is considered statistically significant (shown in bold)

TSRH patients were more likely to have a positive ANA (74% vs. 55%, *p* = 0.0099, SMD = 0.41) but no other significant lab differences were observed. No differences were found for use of NSAIDs or non-biologic DMARDs at the time of injection. However, significantly more children at CHOP were on biologics (8% vs. 1%, *p* = 0.0432, SMD = 0.35) and had additional (2+) joints injected (other than knee) (47% vs. 20%, *p* = 0.0002, SMD = 0.60). The calculated knee injection dose was higher at CHOP (0.43 mg/kg vs. 0.27 mg/kg at TSRH) but this did not reach statistical significance (*p* = 0.26, SMD = 0.19).

In terms of outcomes, there was a trend toward a higher recurrence rate of arthritis in the injected knee at TSRH (38% vs 26%, *p* = 0.14, SMD = 0.25) Time to recurrent arthritis did not differ between institutions (~ 10–10.5 months, Table 3).

**Table 3:** (Final/Follow-up) Clinical Characteristics, N = 167 (single injection)

N(%) or Median (25th–75th)					
	All (N = 167)	CHOP (N = 72)	TSRH (N = 95)	P-value*	SMD
Joint swelling					
> 1 (vs 0)	48 (29%)	15 (21%)	33 (35%)	**0.0493**	**0.31**
Joint tenderness					
> 1 (vs 0)	9 (5%)	6 (8%)	3 (3%)	0.18	0.22
Joint limited range of motion					
> 1 (vs 0)	33 (20%)	7 (10%)	26 (27%)	**0.0046**	**0.47**
Arthritis reoccurrence – Yes	55 (33%)	19 (26%)	36 (38%)	0.14	0.25
If yes, number of months until arthritis reoccurred	10 (4–19)	10 (4–19)	10.5 (4–18)	0.87	0.05
CHAQ Score Follow-up^a^	0.125 (0–0.50)	0.125 (0–0.125)	0.125 (0–0.50)	0.26	0.20
MD Global Score Follow-up^b^	0 (0–1)	0 (0–1)	0 (0–1)	0.88	0.03
Pain Assessment Follow-up (1–10)	0 (0–3)	0 (0–3.5)	1(0–3)	0.90	0.02

*Wilcoxon Rank-Sum tests or Chi-squared/Fisher’s Exact Tests between CHOP and TSRH sites, alpha = 0.05

^a^CHAQ missing, *n* = 34

^b^MD Global missing, n = 30

^c^Pain assessment missing, *n* = 36

SMD: standardized mean difference. SMD > 0.25 (25%) indicates an imbalance between groups, *P* < 0.05 is considered statistically significant (shown in bold)

Cox proportional Hazards regression demonstrates risk of 1.15 (unadjusted) for arthritis reoccurrence at TSRH vs CHOP (Table 4). Propensity score weights were built as previously described. Additional file 1: Figure S1 shows the reduction of imbalance of covariates when comparing weighted and unweighted absolute standardized differences. Using these weights, an IPTW-adjusted was run and adjusted hazards ratio between TSRH vs. CHOP was 1.26 (95% CI 0.55–2.90), which was still not statistically significant. Balance between the two sites was assessed using a number of diagnostic criteria including pre-post weighted SMD comparisons (Additional file 1: Figure S1 & Additional file 3: Table S1). Additional file 2: Figure S2 shows the weighted Kaplan-Meier survival curves between the two sites with significant overlap. In the first 6 months after injection, TSRH patients had a significantly increased risk of reoccurrence vs CHOP, but following then, it was no longer significant.

**Table 4:** Cox Proportional Hazards Regression Models for Arthritis Reoccurrence by Site

	Hazard Ratio - Exp(β) (95% CI)	P-value
Model 1 – Unadjusted		
TSRH (vs. CHOP)	1.15 (0.66-2.01)	0.62
Model 2 - Propensity Score Adjustment*		
TSRH (vs. CHOP)	1.26 (0.55-2.90)	0.58

*Inverse probability of treatment weight using propensity scores. Propensity scores were estimated using TWANG SAS Macros (Griffin et al, RAND Corp. 2014) for group differences between CHOP and TSRH (adjusted for joint swelling, joint tenderness, joint range of motion, knee injection dose, ANA status, age at diagnosis, and race). Cox model additionally adjusted for disease duration, total joints active and biologics injected since the effect sizes between sites were still >0.25 after PS balancing.

Additional analyses of this cohort were completed to determine if there were any differences in baseline demographic or clinical characteristics between those with or without recurrent arthritis of the injected knee during the follow-up period. There were no differences in the baseline demographic data (Table 5). However, there were some notable differences in baseline clinical characteristics (Table 6). Patients with recurrent arthritis during the follow-up period had more significant joint swelling and limited range of motion at baseline (*p* < 0.5). Although there were no differences in systemic medication use, there was a trend to lower knee injection dose (mg/kg) in those with recurrent arthritis (not statistically significant). The number of joints injected (in addition to the knee) did not impact outcome.

**Table 5:** Population Characteristics, *N* = 167 (single injection) stratified by Arthritis Reoccurrence Status

N(%) or Median (25th–75th)					
	All (N = 167)	No Reoccurrence of Arthritis (*N* = 112)	Arthritis Reoccurrence (*N* = 55)	*P*-value*	SMD
Sex – Female	136 (81%)	92 (82%)	44 (80%)	0.74	0.05
Age, year	6.7 (3.8–10.5)	6.6 (3.6–10.4)	7.1 (4.0–10.6)	0.84	0.03
Race				0.40	0.22
Black/AA	5 (3%)	2 (2%)	3 (5%)		
White	149 (90%)	100 (90%)	49 (89%)		
Other	12 (7%)	9 (8%)	3 (5%)		
White (vs. nonwhite)	149 (90%)	100 (90%)	49 (89%)	0.97	0.01
Ethnicity – Hispanic (vs not)	25 (15%)	17 (15%)	8 (15%)	0.88	0.02
Symptom Age Start	4.6 (2.3–8.7)	4.8 (2.5–8.9)	4.1 (2.2–8.2)	0.50	0.13
Knee Symptom Age Start	5.6 (3.2–9.7)	5.6 (3.1–9.6)	5.7 (3.3–10.3)	0.50	0.13
Diagnosis age	4.0 (2.5–6.8)	4.2 (2.5–6.9)	3.9 (2.6–6.0)	0.61	0.09
Systolic Blood Pressure	106.0 (101.0–115.5)	106.0 (99.0–115.0)	106.5 (102.0–116.0)	0.31	0.25
Diastolic Blood Pressure	63.0 (57.0–66.0)	62.0 (57.0–66.0)	64.0 (59.0–66.0)	0.17	0.24
Iritis – Yes*	4 (4%)	2 (3%)	2 (5%)	0.61	0.13

*Wilcoxon Rank-Sum tests or Chi-squared/Fisher’s Exact Tests between Arthritis reoccurrence 1 vs 0, alpha = 0.05

**Iritis data, n = 70 missing (36%)

SMD: standardized mean difference

**Table 6:** Clinical Characteristics, N = 167 (single injection)

N(%) or Median (25th–75th)					
	All (N = 167)	No Reoccurrence of Arthritis (N = 112)	Arthritis Reoccurrence (N = 55)	P-value*	SMD
Joint swelling					
> 1 (vs 0)	161 (96%)	109 (97%)	52 (95%)	0.40	0.14
Joint tenderness					
> 1 (vs 0)	30 (18%)	20 (18%)	10 (18%)	0.96	0.01
Joint limited range of motion					
> 1 (vs 0)	95 (57%)	66 (59%)	29 (53%)	0.45	0.13
Total joints active				0.99	0.21
1	90 (54%)	59 (53%)	31 (56%)		
2	58 (35%)	39 (35%)	19 (35%)		
3	13 (8%)	9 (8%)	4 (7%)		
4	4 (2%)	3 (3%)	1 (2%)		
5	0	2 (2%)	0		
6	2 (1%)				
2+ Joints Active (vs 1 only)	77 (46%)	53 (47%)	24 (44%)	0.65	0.07
Pain Assessment (1–10)^a^	1 (0–4)	1.5 (0–4.0)	1 (0–3)	0.88	0.03
CHAQ Score^b^	0.125 (0–0.50)	0.125 (0–0.500)	0 (0–0.313)	0.07	0.45
MD Global Score^c^	1(1–2)	2 (1–2)	1 (1–2)	0.11	0.36
ANA status–Positive	108 (66%)	68 (62%)	40 (74%)	0.12	0.26
RF status– Positive	4 (3%)	4 (4%)	0	0.30	0.29
HLA-B27 status–Positive	14 (13%)	7 (11%)	7 (17%)	0.36	0.18
ESR^d^	14 (8–23)	12 (8–20)	16 (8–26)	0.40	0.16
On injection DMARDs	29 (17%)	19 (17%)	10 (18%)	0.85	0.03
On injection biologics	7 (4%)	6 (5%)	1 (2%)	0.43	0.19
On NSAIDs	109 (65%)	71 (63%)	38 (69%)	0.47	0.12
Total joints injected				0.87	0.03
2+	53 (32%)	36 (32%)	17 (31%)		
1	114 (68%)	76 (68%)	38 (69%)		
Steroid injection - Aristopan (vs. Kenalog)	156 (94%)	107 (96%)	49 (89%)	0.08	0.24
Knee injection dose (mg)	10 (2–20)	10 (2–20)	10 (2–20)	0.33	0.16
Knee injection dose (mg/kg)^d^	0.33 (0.10–0.78)	0.40 (0.10–0.77)	0.19 (0.10–0.84)	0.50	0.12

*Wilcoxon Rank-Sum tests or Chi-Square/Fisher’s Exact Tests between arthritis reoccurrence yes/no, alpha = 0.05

^a^Pain Assessment is missing for 42% (n = 70) of the data (46% CHOP; 39% TSRH)

^b^CHAQ score missing for 56% (n = 94) of the data (81% CHOP; 38% TSRH)

^c^MD Global score missing for 46% (n = 77) of the data (74% CHOP; 25% TSRH)

^d^ESR score missing for 31% (n = 52) of the data

SMD: standardized mean difference

## Discussion

To our knowledge, this is the first study to examine post-IACI practices in patients with JIA. Although there was a trend toward a higher arthritis recurrence rate at the center with post-injection joint splinting (TSRH), this did not reach statistical significance. Of note there were significant differences in the populations of patients at CHOP vs TSRH. When controlling for baseline differences in patient populations, including age of disease onset, race, ANA positivity, baseline joint assessment severity, and knee injection dose, the risk for recurrent arthritis did not differ between the two institutions. In addition, the average time to recurrent arthritis was comparable. Therefore, application of a splint, along with instructions to rest the joint for 24 to 48 h post-IACI, may not provide additional benefit. However, this was a retrospective study and we cannot determine if patients were compliant with splint use. We also could not examine the impact of splinting on the occurrence of adverse events, particularly dermal or lipo-atrophy at the injection site.

When examining the entire cohort, there were a few baseline differences between those with and without recurrent arthritis during the follow-up period. However, there was more recurrent arthritis when the knee was more severely affected at the time of injection. It is unknown whether the knee had ever been previously injected. There is evidence that injection of a joint, including the knee, earlier in the disease course may positively affect outcomes, including protracted response [11]. Factors previously found to impact positively IACI outcomes were not seen in this cohort, including more elevated ESR [10], younger age at diagnosis, and presence of uveitis [35]. The average time to recurrent arthritis in this cohort, 10 months, is shorter than that seen in recent studies: 12.5 months [36] and 18.1 months [35]. Although the previously mentioned studies examined the outcome of a variety of joint injections, most were knees.

The majority of subjects were treated with triamcinolone hexacetonide (Aristospan®), known to have a longer duration of clinical response [16, 19]. However, the average dose for this cohort was 0.33 mg/kg (range 0.1 to 0.78). This may have shortened the time to recurrent arthritis as several studies indicate that the optimal dose should be between 1 to 1.5 mg/kg [36, 37]. There was a trend toward a lower average mg/kg dose in those that had recurrent arthritis during the follow-up period.

Since there is some evidence from the adult literature that a specific post-IACI protocol may impact outcomes, particularly for knee injections [26, 27] which are quite common in JIA, it would be beneficial to study this question in additional cohorts. Future studies should be conducted in a prospective manner and potentially include several treatment arms (post-IACI protocols) including resumption of normal activity, prescribed period of rest, and immobilization. Patient adherence should be monitored since rest and immobilization is hard to ensure in young children. It will also be important to examine other secondary outcomes such as such as range of motion, leg length discrepancy, pain, function and adverse events. Although there were differences in CHAQ, pain, and MD Global scores between the two patient populations at baseline, there was a large amount of missing data. Certainly, this was a major limitation of this retrospective study. Including pre and post-imaging studies, such as ultrasound, would allow for objective measures of baseline joint severity and response. Finally, it will be important to standardize the injection dose, considering whether a mg/kg dose is the best approach.

## Conclusions

There was not a statistically significant risk for recurrent arthritis in the group that was not splinted versus splinted after IACI of the knee. In patients with recurrent arthritis, there was no significant difference in the splinted group and the non-splinted group (other factors being controlled). Based upon our study, we cannot definitively recommend splinting knees post-IACI for oligoarticular JIA patients. This is important for oligoarticular JIA patients who have had IACI’s as it will save the time, effort, and expense of splinting.

## Additional files

Additional file 1: **Figure S1.** Standardized Effect Sizes Pre/Post Weighting using TWANG macro. (DOCX 45 kb)

Additional file 2: **Figure S2.** Weighted Kaplan Meier Curve: Arthritis Reoccurrence. (DOCX 33 kb)

Additional file 3: **Table S1.** Standardized Effect Sizes Pre/Post Weighting using TWANG macro. (DOCX 12 kb)

## Abbreviations

ATE: average treatment effect
CHAQ: Childhood Health Assessment Questionnaire
CHOP: The Children’s Hospital of Philadelphia
DMARD: disease-modifying antirheumatic drugs
IACI: intra-articular corticosteroid injection
ILAR: International League of Associations for Rheumatology
IPTW: inverse probability of treatment weighting
JIA: Juvenile Idiopathic Arthritis
NSAID: non-steroidal anti-inflammatory drugs
ROM: range of motion
SMD: standardized mean differences
TSRH: Texas Scottish Rite Hospital for Children
