# Supplementary figures and images for: Retrospective review of immobilization vs. immediate resumption of activity in patients with Oligoarticular juvenile idiopathic arthritis following knee injections
Source: Pediatr Rheumatol Online J. 2019 Jul 12;17:42. doi: 10.1186/s12969-019-0339-0 (PMC6626321; doi:10.1186/s12969-019-0339-0)

Additional file 1: Figure S1. Standardized Effect Sizes Pre/Post Weighting using TWANG macro


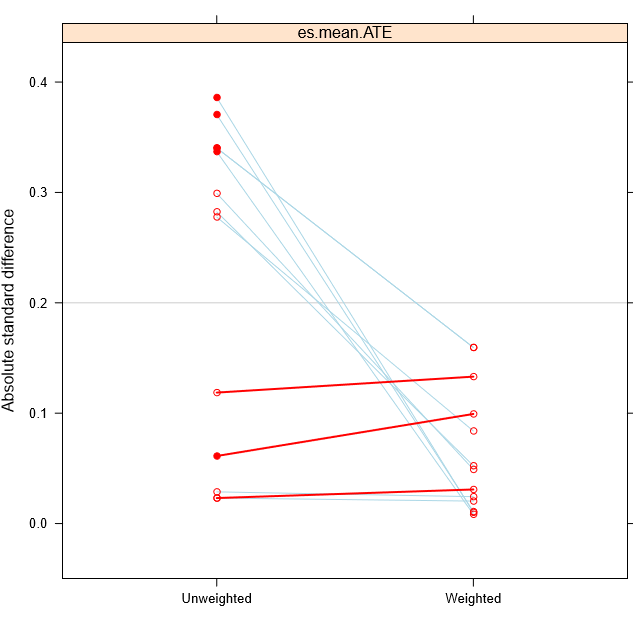

Supplement: Supplementary file 1 — Figure S1. Standardized Effect Sizes Pre/Post Weighting using TWANG macro. (DOCX 45 kb) [file 12969_2019_339_MOESM1_ESM.docx]
