# Supplementary material for: Retrospective review of immobilization vs. immediate resumption of activity in patients with Oligoarticular juvenile idiopathic arthritis following knee injections
Source: Pediatr Rheumatol Online J. 2019 Jul 12;17:42. doi: 10.1186/s12969-019-0339-0 (PMC6626321; doi:10.1186/s12969-019-0339-0)
Supplement: Supplementary file 2 — Figure S2. Weighted Kaplan Meier Curve: Arthritis Reoccurrence. (DOCX 33 kb) [file 12969_2019_339_MOESM2_ESM.docx]

Additional file 2: Figure S2. Weighted Kaplan Meier Curve: Arthritis Reoccurrence


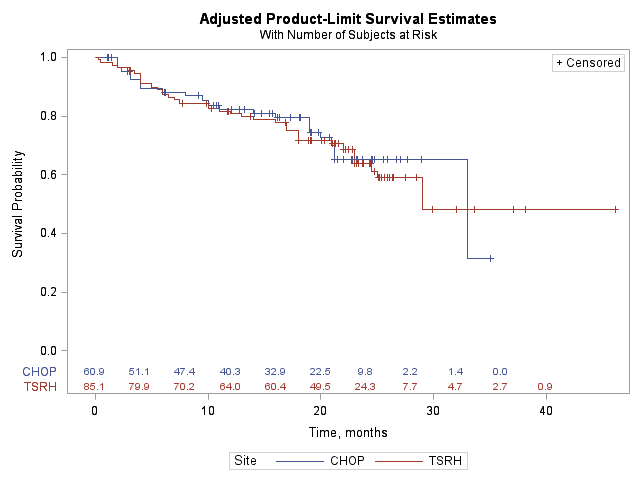


Time in months

CHOP-Children’s Hospital of Philadelphia

TSRH-Texas Scottish Rite Hospital
