# Supplementary material for: Retrospective review of immobilization vs. immediate resumption of activity in patients with Oligoarticular juvenile idiopathic arthritis following knee injections
Source: Pediatr Rheumatol Online J. 2019 Jul 12;17:42. doi: 10.1186/s12969-019-0339-0 (PMC6626321; doi:10.1186/s12969-019-0339-0)
Supplement: Supplementary file 3 — Table S1. Standardized Effect Sizes Pre/Post Weighting using TWANG macro. (DOCX 12 kb) [file 12969_2019_339_MOESM3_ESM.docx]

Appendix 2, Table 1. Standardized Effect Sizes Pre/Post Weighting using TWANG macro

|  | Absolute Standardized Difference | |
| --- | --- | --- |
| Covariate | Unweighted | Weighted |
| Race - Black | 0.023 | 0.031 |
| Race - White | 0.278 | 0.084 |
| Race - Other | 0.282 | 0.052 |
| Race - Missing | 0.119 | 0.133 |
| Joint Range of Motion - 0 | 0.340 | 0.160 |
| Joint Range of Motion - 1 | 0.340 | 0.160 |
| Ana Status - None | 0.386 | 0.010 |
| Ana Status - Present | 0.371 | 0.011 |
| Ana Status -Missing | 0.061 | 0.010 |
| Age at Diagnosis | 0.337 | 0.008 |
| Age at Diagnosis - Missing | 0.023 | 0.020 |
| Knee Dose | 0.299 | 0.049 |
| Knee Dose - Missing | 0.029 | 0.024 |
